# The *Acinetobacter baumannii* website (Ab-web): a multidisciplinary knowledge hub, communication platform, and workspace

**DOI:** 10.1093/femsmc/xtad009

**Published:** 2023-04-21

**Authors:** Nabil Karah, Valeria Mateo-Estrada, Santiago Castillo-Ramírez, Paul G Higgins, Benjamin Havenga, Wesaal Khan, Sara Domingues, Gabriela Jorge Da Silva, Laurent Poirel, Patrice Nordmann, Cecilia Ambrosi, Chaoying Ma, Siobhán McClean, María Paula Quiroga, Verónica E Alvarez, Daniela Centron, Raffaele Zarrilli, Johanna J Kenyon, Thomas A Russo, Benjamin A Evans, Andres Opazo-Capurro, Rayane Rafei, Monzer Hamze, Ziad Daoud, Irfan Ahmad, Philip N Rather, Ruth M Hall, Gottfried Wilharm, Bernt Eric Uhlin

**Affiliations:** Department of Molecular Biology and Umeå Centre for Microbial Research (UCMR), Umeå University, 901 87 Umeå, Sweden; Department of Research and Development, KARAHCO, 0951 Oslo, Norway; Programa de Genómica Evolutiva, Centro de Ciencias Genómicas, Universidad Nacional Autónoma de México, 62210 Cuernavaca, México; Programa de Genómica Evolutiva, Centro de Ciencias Genómicas, Universidad Nacional Autónoma de México, 62210 Cuernavaca, México; Institute for Medical Microbiology, Immunology and Hygiene, Faculty of Medicine and University Hospital Cologne, University of Cologne, 50935 Cologne, Germany; German Center for Infection Research (DZIF), Partner Site Bonn-Cologne, D-50935 Cologne, Germany; Department of Microbiology, Faculty of Science, Stellenbosch University, Private Bag X1, 7602 Stellenbosch, South Africa; Department of Microbiology, Faculty of Science, Stellenbosch University, Private Bag X1, 7602 Stellenbosch, South Africa; Faculty of Pharmacy, University of Coimbra, 3000-458 Coimbra, Portugal; Center for Neuroscience and Cell Biology, University of Coimbra, 3004-517 Coimbra, Portugal; Faculty of Pharmacy, University of Coimbra, 3000-458 Coimbra, Portugal; Center for Neuroscience and Cell Biology, University of Coimbra, 3004-517 Coimbra, Portugal; Medical and Molecular Microbiology, Department of Medicine, Faculty of Science and Medicine, University of Fribourg, Chemin du Musée 18, 1700 Fribourg, Switzerland; Swiss National Reference Center for Emerging Antibiotic Resistance (NARA), University of Fribourg, 1700 Fribourg, Switzerland; Medical and Molecular Microbiology, Department of Medicine, Faculty of Science and Medicine, University of Fribourg, Chemin du Musée 18, 1700 Fribourg, Switzerland; Swiss National Reference Center for Emerging Antibiotic Resistance (NARA), University of Fribourg, 1700 Fribourg, Switzerland; Department of Human Sciences and Promotion of the Quality of Life, San Raffaele Open University, IRCCS, 00166 Rome, Italy; School of Biomolecular and Biomedical Sciences, University College Dublin, Belfield D04 V1W8, Dublin 4, Ireland; School of Biomolecular and Biomedical Sciences, University College Dublin, Belfield D04 V1W8, Dublin 4, Ireland; Laboratorio de Investigaciones en Mecanismos de Resistencia a Antibióticos, Instituto de Investigaciones en Microbiología y Parasitología Médica, Facultad de Medicina, Universidad de Buenos Aires - Consejo Nacional de Investigaciones Científicas y Tecnológicas (IMPaM, UBA-CONICET), 1245 Ayacucho (C1111AAI), Buenos Aires, Argentina; Laboratorio de Investigaciones en Mecanismos de Resistencia a Antibióticos, Instituto de Investigaciones en Microbiología y Parasitología Médica, Facultad de Medicina, Universidad de Buenos Aires - Consejo Nacional de Investigaciones Científicas y Tecnológicas (IMPaM, UBA-CONICET), 1245 Ayacucho (C1111AAI), Buenos Aires, Argentina; Laboratorio de Investigaciones en Mecanismos de Resistencia a Antibióticos, Instituto de Investigaciones en Microbiología y Parasitología Médica, Facultad de Medicina, Universidad de Buenos Aires - Consejo Nacional de Investigaciones Científicas y Tecnológicas (IMPaM, UBA-CONICET), 1245 Ayacucho (C1111AAI), Buenos Aires, Argentina; Department of Public Health, University of Naples Federico II, 80138 Naples, Italy; Centre for Immunology and Infection Control, School of Biomedical Sciences, Faculty of Health,, Queensland University of Technology, Brisbane City QLD 4000, Australia; Veterans Administration Western NY, Healthcare System, epartment of Medicine, Jacobs School of Medicine and Biomedical Sciences, University Buffalo, Buffalo, NY 14260, United States; Norwich Medical School, University of East Anglia, Norwich NR4 7TJ, United Kingdom; Laboratorio de Investigación en Agentes Antibacterianos, Departamento de Microbiología, Facultad de Ciencias Biológicas, Universidad de Concepción, 4070386 Concepción, Chile; Laboratoire Microbiologie Santé et Environnement, Doctoral School of Sciences and Technology, Faculty of Public Health, Lebanese University, Tripoli 1300, Lebanon; Laboratoire Microbiologie Santé et Environnement, Doctoral School of Sciences and Technology, Faculty of Public Health, Lebanese University, Tripoli 1300, Lebanon; College of Medicine, Central Michigan University, Mount Pleasant, MI 48859, United States; Department of Clinical Microbiology, Michigan Health Clinics, Saginaw, MI 48604, United States; Department of Molecular Biology and Umeå Centre for Microbial Research (UCMR), Umeå University, 901 87 Umeå, Sweden; Institute of Biomedical and Allied Health Sciences, University of Health Sciences, Lahore, Punjab 54600, Pakistan; Department of Microbiology and Immunology, Emory University School of Medicine, Atlanta, GA 30307, United States; Research Service, Department of Veterans Affairs, Atlanta Veterans Affairs (VA) Medical Center, Decatur, GA 30033, United States; School of Life and Environmental Sciences, The University of Sydney, Sydney, NSW 2006, Australia; Robert Koch Institute, Project Group P2 (*Acinetobacter baumannii*—Biology of a Nosocomial Pathogen), Burgstr 37, 38855 Wernigerode, Germany; Department of Molecular Biology and Umeå Centre for Microbial Research (UCMR), Umeå University, 901 87 Umeå, Sweden

**Keywords:** online educational platform, clinical microbiology, antimicrobial resistance, virulence, molecular epidemiology, mobile genetic elements

## Abstract

*Acinetobacter baumannii* is a Gram-negative bacterium increasingly implicated in hospital-acquired infections and outbreaks. Effective prevention and control of such infections are commonly challenged by the frequent emergence of multidrug-resistant strains. Here we introduce Ab-web (https://www.acinetobacterbaumannii.no), the first online platform for sharing expertise on *A. baumannii*. Ab-web is a species-centric knowledge hub, initially with 10 articles organized into two main sections, ‘Overview’ and ‘Topics’, and three themes, ‘epidemiology’, ‘antibiotic resistance’, and ‘virulence’. The ‘workspace’ section provides a spot for colleagues to collaborate, build, and manage joint projects. Ab-web is a community-driven initiative amenable to constructive feedback and new ideas.

## Teaser

Ab-web is a community-driven initiative seeking to share knowledge, expertise, and research ideas about *Acinetobacter baumannii*, an emerging and remarkably resilient opportunistic pathogen.

## Introduction

*Acinetobacter baumannii* is a non-spore-forming, glucose-non-fermenting, rod-shaped Gram-negative bacterium that has, over the last four decades, become a notorious opportunistic pathogen. It belongs to the ESKAPE group of pathogens (*Enterococcus faecium, Staphylococcus aureus, Klebsiella pneumoniae, A. baumannii, Pseudomonas aeruginosa*, and *Enterobacter* spp.) known to exhibit a range of virulence and multidrug resistance (MDR) features, enabling them to evade or ‘escape’ commonly used antibiotics (Boucher et al. 2009). Importantly, carbapenem-resistant *A. baumannii* has recently been ranked as ‘Priority 1: CRITICAL’ in the World Health Organization list of bacteria for which new antibiotics are urgently needed (Tacconelli et al. 2018).

Although the emergence of highly virulent strains of *A. baumannii* has been reported (Singh et al. 2020), our knowledge of the mechanisms of *A. baumannii* pathogenicity is still limited, pending further research. The remarkable abilities of *A. baumannii* to persist on dry surfaces, to acquire resistance to different classes of antibiotics, and to effectively disseminate within and among medical facilities have been underlined (Sarshar et al. 2021). The global epidemiology of *A. baumannii*, though it is currently highly biassed by a large number of clinical isolates and much fewer veterinary or environmental strains, is mainly dominated by a few epidemic clones, especially international clones (IC) 1 and 2, also known as global clones (GC) 1 and 2 (Higgins et al. 2017, Gaiarsa et al. 2019). IC7, which corresponds to sequence types (ST) 25 and 229 according to the Pasteur (p) and Oxford (ox) multilocus sequence typing (MLST) schemes, respectively, is another worldwide prevalent clone (Sahl et al. 2015). In addition, IC5 (ST79^P^ and ST205^Ox^) and IC4 (ST15^p^ and ST438^Ox^) are major epidemic clonal lineages in South America (Brito et al. 2022).

The high adaptability of these clones to hospital settings has been attributed to their antibiotic resistance and the expression of multiple virulence and resilience factors, including cellular envelope and outer membrane proteins, secretion systems, iron acquisition systems, and phospholipases (Harding et al. 2018). Plasmids and other mobile genetic elements (MGEs) have been key contributors to the evolution of such extremely successful clones of *A. baumannii*. For instance, plasmid R16, first reported in 1956, contributed to the formation of a resistance island that has become a stable structure in the chromosome of *A. baumannii* IC1 (Moran and Hall 2019). Another recent study has demonstrated the capacity of pAB5, a large conjugative plasmid, to impact the expression of chromosomally encoded virulence factors and to confer niche specificity to *A. baumannii* uropathogenic strains (Di Venanzio et al. 2019).

While the earliest articles on *A. baumannii* were published in the 1940s, though under different names, the official name of this species was coined in 1986 (Bouvet and Grimont 1986). Since then, a growing body of literature has accumulated, including >12 400 entries found in the PubMed database for the search query ‘*Acinetobacter baumannii*’ (https://pubmed.ncbi.nlm.nih.gov/?term = acinetobacter+baumannii; as of 22 January 2023). Notably, free full text is available for only 55% of these results. Similarly, mounting records of *A. baumannii* sequence data are deposited in GenBank on a daily basis, including more than 17 500 assemblies, more than 7380 ‘Genome Assembly and Annotation’ reports, and 980 ‘Plasmid Annotation’ reports as of 22 January 2023 (https://www.ncbi.nlm.nih.gov/assembly/?term= acinetobacter±baumannii; https://www.ncbi.nlm.nih.gov/genome/?term = acinetobacter%20baumannii). Nevertheless, researchers are challenged by the lack of an existing focal repository point for accessing and sharing data on *A. baumannii* and by a noticeable shortage in the number and effectiveness of freely accessible sequence analysis servers tailored to meet the specificities of different bacterial species.

Here, we present the development of the first *A. baumannii* website (Ab-web) as a free online multidisciplinary knowledge hub aiming to centralize information on *A. baumannii*. Ab-web offers a platform for knowledge exchange, capacity development, and coordination to research groups, health networks, institutes, authorities, infrastructures, and corporations involved in the study of *A. baumannii* all over the world. Ab-web provides access to a collection of *in-house* expert-written and peer-reviewed web pages on the biological features, ecology, evolution dynamics, host interactions, and epidemiology of infections caused by *A. baumannii*. Hosting sequence analysis services tailored to *A. baumannii* will be one of our main goals in the short term.

### Website structure and contents

The three main ‘Overview’ web pages summarize current knowledge on antibiotic resistance, global epidemiology, and the virulence of *A. baumannii*. One sub-page, arranged under the ‘Global Epidemiology’ page, is dedicated to exploring the epidemiology of *A. baumannii* IC1 (Holt et al. 2016, Higgins et al. 2017). The six ‘Topics’ web pages provide insights into (i) the nomenclature, evolution, and clinical role of the *A. baumannii*-intrinsic gene *oxaAb* (also known as *bla*_OXA-51-like_) (Takebayashi et al. 2021); (ii) importance, structural diversity, and biosynthesis of the *A. baumannii* polysaccharide capsule (Russo et al. 2010, Kenyon and Hall 2013); (iii) mechanisms of horizontal gene transfer and natural transformation in *A. baumannii* (Wilharm and Skiebe 2019); (iv) genetic structure of Tn*6019*, the backbone transposon of AbaR3-type resistance islands in *A. baumannii* (Post et al. 2010); (v) phase variation switch between opaque and translucent colony phenotypes (Anderson and Rather 2019); and (vi) hospital and extra-hospital epidemiology of *A. baumannii* in Lebanon (Dandachi et al. 2019). The latter topic is made as a model to encourage other colleagues to present the epidemiological story of *A. baumannii* in their countries. Such country-wise sub-pages can later be merged into one large chapter.

All the web pages will be regularly edited and updated. Both the authors and reviewers have access to the website builder and full self-control to edit the web pages. Ab-web is hosted by one.com and can be accessed via any internet browser (https://www.acinetobacterbaumannii.no). It offers information to a variety of audiences from students, researchers, healthcare staff, and policymakers to affected individuals and the general public. All users have straightforward access to all the published material in the form of texts, tables, and figures. Users are free to download high-resolution figures of the MGE maps and re-use the data as long as attribution is provided. Registering, free of charge, is needed to become a member of the Ab-web community, allowing registered users to get involved in discussion groups and workspace activities. As a pilot project, this manuscript was prepared using a workspace that was open to all the co-authors. A joint study on the phenotypic and genotypic features of *A. baumannii* IC1 lineage 3, first reported in 2021 (Hamidian and Hall 2021), is currently under progress in the workspace section. Some of the discussion groups and workspaces will be made ‘open’ and will be visible to all the Ab-web visitors to facilitate wider engagement. Ab-web will be governed by a steering committee composed of four or five experts in the field of *A. baumannii*. The main tasks of the steering committee are to ensure adherence to the code of conduct for responsible research (e.g. https://www.who.int/about/ethics/code-of-conduct-for-responsible-research) and compliance with common guidelines for research website usability and best practice in scholarly publishing (e.g. https://publicationethics.org/resources/guidelines-new/principles-transparency-and-best-practice-scholarly-publishing), and to create plans and provide guidance on the development of the website.

The digital object identifiers (DOIs) of all the reference articles and original studies are hyperlinked to the websites of their publishers. Similarly, links are generated to direct users to other relevant online databases and tools, such as PubMLST (https://pubmlst.org/organisms/acinetobacter-baumannii), PubMed (https://pubmed.ncbi.nlm.nih.gov/), GenBank (https://www.ncbi.nlm.nih.gov/genbank/), PathogenWatch (https://pathogen.watch/genomes/all?genusId = 469), and Kaptive (https://kaptive-web.erc.monash.edu/).

### Plans for future developments

The hub will continue to be a community-driven, collaborative initiative. Feedback and suggestions will be deemed and applied to improve the functionality of Ab-web. The multiplicity of our informative web pages will be steadily expanded. Once approved by the steering committee, registered users will have direct access to create new pages. For example, the need to add a page on the clinical aspects and current therapies of *A. baumannii* infections has already been noted. Adding a web page to highlight recent developments and evaluations of new therapeutic drugs, such as cefiderocol and durlobactam (Isler et al. 2018), is also needed. Likewise, web pages on the epidemiology of *A. baumannii* IC2, the global distribution of colistin-resistant isolates, biofilm formation, the type VI secretion system, and bacteriophages are missing and shall be added. A practical guideline on antimicrobial susceptibility testing of *A. baumannii* shall be retrieved from reference resources such as the Clinical and Laboratory Standards Institute—CLSI (https://clsi.org/) and the European Committee on Antimicrobial Susceptibility Testing—EUCAST (https://www.eucast.org/). Considering that antibiotic-resistant *A. baumannii* is a One Health problem (Hernández-González and Castillo-Ramírez 2020), an overview webpage will be created to summarize our current understanding of the non-human epidemiology of *A. baumannii*. Web pages related to studies interconnecting data from people, animals, plants, and the environment will also be encouraged.

The Ab-web platform shall gradually provide access to additional assets, such as trending news and events, protocols and standard operating procedures, links to relevant workshops, symposiums and conferences, surveillance data, annotated sequences, and bioinformatic services. The annotation of genetic elements deposited in the commonly used sequence databases is often incomplete, and information about the presence, location, or precise identity of some of the genetic features might sometimes be missing (Ross et al. 2021). One of our future aims is to integrate web applications allowing users to upload new sequences, generate initial automatic annotations, facilitate manual revisions, and cooperate to build peer-curated genetic maps.

### Database of the *A. baumannii* MGEs

To recognize the impact of MGEs on the evolution of *A. baumannii*, there is a need to create and maintain a user-friendly catalogue of all detected MGEs. Ab-web can be enhanced by creating specifically designed web page(s) to function as an interface for a carefully annotated and expert-reviewed database of the *A. baumannii* MGE transposition genes, accessory genes, antimicrobial or heavy metal resistance genes, other functional genes, and other core or non-core genetic sequences or elements. The catalogue shall provide a detailed description of the MGEs, where each MGE shall have its own entry page, including a detailed table and graphical map of their genetic structures. The website will be equipped with a nucleotide similarity search engine, similar to the Basic Local Alignment Search Tool (https://blast.ncbi.nlm.nih.gov/Blast.cgi), tool for genetic map construction, such as SnapGene (https://www.snapgene.com/), a filtering algorithm to display only the manually curated feature matches, and an application to visualize comparisons between multiple entries, such as Easyfig (http://mjsull.github.io/Easyfig/), all of which can be used by scientists to initiate more studies on the source and provenance of newly detected MGEs and those of particular interest.

## Conclusion

The frequent emergence of multidrug-resistant strains of *A. baumannii* has raised the concern of lacking effective treatment options. Our Ab-web knowledge hub is the first online species-centric library for *A. baumannii*, with a compilation of 10 peer-reviewed articles on its virulence, epidemiology, and antibiotic resistance features. Ab-web facilitates straightforward associations with colleagues and experts, offering a virtual platform to plan, develop, and deploy new projects. While promoting research on *A. baumannii* is our main goal, Ab-web shall also inform optimal management of infections caused by this difficult-to-treat opportunistic pathogen, promote timely response to outbreaks, and improve patient outcomes. Ab-web also has the potential of becoming a portal for genomic analyses of *A. baumannii*, with a variety of basic research and applied clinical applications.
